# Developmental, Dietary, and Geographical Impacts on Gut Microbiota of Red Swamp Crayfish (*Procambarus clarkii*)

**DOI:** 10.3390/microorganisms8091376

**Published:** 2020-09-08

**Authors:** Zhenting Zhang, Jiali Liu, Xuexia Jin, Chao Liu, Chenwei Fan, Li Guo, Yunxiang Liang, Jinshui Zheng, Nan Peng

**Affiliations:** 1 State Key Laboratory of Agricultural Microbiology, College of Life Science and Technology, Huazhong Agricultural University, Wuhan 430070, China; zhangzhenting@webmail.hzau.edu.cn (Z.Z.); ljl29@webmail.hzau.edu.cn (J.L.); jinxuexia1127@webmail.hzau.edu.cn (X.J.); fancw96@163.com (C.F.); fa-lyx@163.com (Y.L.); 2 Runge College of Bioengineering, Sichuan Runge Biotechnology Co., Ltd., Mianzhu, Deyang 618200, China; 13545085590@163.com; 3 State Key Laboratory of Electrical Insulation and Power Equipment, Center for Plasma Biomedicine, Xi’an Jiaotong University, Xi’an 710049, China; guoli35@mail.xjtu.edu.cn; 4 State Key Laboratory of Agricultural Microbiology, College of Informatics, Huazhong Agricultural University, Wuhan 430070, China; jszheng@mail.hzau.edu.cn

**Keywords:** red swamp crayfish, gut microbiota, shaping factors, fermented feed, high-throughput sequencing, *Procambarus clarkii*

## Abstract

Red swamp crayfish (*Procambarus clarkii*) breeding is an important economic mainstay in Hubei province, China. However, information on the gut microbiota of the red swamp crayfish is limited. To address this issue, the effect of developmental stage, diet (fermented or non-fermented feed), and geographical location on the gut microbiota composition in the crayfish was studied via high-throughput 16S rRNA gene sequencing. The results revealed that the dominant phyla in the gut of the crayfish were *Proteobacteria*, *Bacteroidetes,* *Firmicutes*, *Tenericutes*, and *RsaHF231*. The alpha diversity showed a declining trend during development, and a highly comparable gut microbiota clustering was identified in a development-dependent manner. The results also revealed that development, followed by diet, is a better key driver for crayfish gut microbiota patterns than geographical location. Notably, the relative abundance of *Bacteroidetes* was significantly higher in the gut of the crayfish fed with fermented feed than those fed with non-fermented feed, suggesting the fermented feed can be important for the functions (e.g., polysaccharide degradation) of the gut microbiota. In summary, our results revealed the factors shaping gut microbiota of the crayfish and the importance of the fermented feed in crayfish breeding.

## 1. Introduction

The key important roles of gut microbiota in various organisms are widely treasured [1]. Primarily, the gut microbiota promotes nutrient absorption and stimulates the immune response and disease resistance in hosts, thus affecting the health status of the individuals [2,3,4,5]. Increasing evidence points out that changes in gut microbial composition are linked to different factors. Previous studies have demonstrated that environmental exposure, diet, immunological pressure, host genetics, and ecological forces within the ecosystem shapes the gut microbial community [6,7,8,9]. Diet is believed to be a key determining factor in shaping gut microbiota in host animals [10]. For instance, diets with different FODMAP (fermentable oligosaccharides, disaccharides, monosaccharides, and polyols) contents have remarkable effects on gut microbiota composition in humans and mice [10]. Moreover, host-associated factors like age and sex can determine the gut microbial community assembly [11]. Many studies have shown that the developmental stage of gut microbiota varies with their variation in human and mice hosts, where they tend to stabilize with increasing age [12]. For some insects, such as the mosquito, the developmental stage and geographical location mainly determine the gut bacterial composition in comparison with the mosquito species or adult feeding status [6]. However, information of shaping factors on the gut microbiota of crayfish is limited.

Furthermore, studying the gut microbiota pattern in breeding animals is a prerequisite for improving the breeding efficiency. Aquatic animals provide high-quality animal protein which improves human nutrition and health [13]. Numerous studies have demonstrated that fish has distinct structures of intestinal bacterial community compared with mammals, suggesting that the host phylogeny shapes the bacterial community of the intestine [7,14,15]. *Litopenaeus vannamei*, also known as white prawn, is the global principal species choice for shrimp aquaculture [16]. However, shrimp aquaculture is threatened by the emergence and spread of diseases, leading to high annual economic losses [17]. According to gut microbial community studies, healthy and diseased shrimp have distinct intestinal bacterial communities [18]. This indicates that the gut microbial community can be used as a potential indicator of shrimp health status [19]. Recent research in aquatic animal has revealed the essential contribution of rearing conditions [20,21], diets [22,23], developmental stage [24,25], health status [18] on the gut microbiota. Compared with mammals, the structure of *L. vannamei* intestinal bacterial is predominantly *Proteobacteria*, with low relative abundances of *Firmicutes* and *Bacteroidetes* [8,12,16].

*Procambarus clakii* (red swamp crayfish) originates from the northeastern Mexico and the south-central United States. It is one of the world’s most invasive species and invaded China from Japan in the 1920s [26]. Invasive species have attracted a considerable amount of attention from conservationists and scientists alike because they potentially pose a threat to native species [27]. Even though the red swamp crayfish is an invasive species, it is an economically important freshwater product in China, especially in Hubei province [28]. This accounts for more than half of crayfish output in the country and is a major source of income for local farmers [28]. Previous studies have applied the next-generation sequencing to describe the gut bacterial communities in Decapods including the European lobster (*Homarus gammarus*) [29], Norway lobster (*Nephrops norvegicus*) [30], cherry shrimp (*Neocaridina denticulate*) [31], Pacific white shrimp (*L. vannamei*) [16,32,33], black tiger shrimp (*Penaeus monodon*) [34] and giant freshwater prawn (*Macrobrachium rosenbergii*) [35]. However, there is limited information on gut bacterial communities of red swamp crayfish (*P. clarkii*). Given the significance of the gut-associated bacteria on the overall health of organisms, we studied the gut microbial communities in *P. clarkii* and factors influencing them.

## 2. Materials and Methods

### 2.1. Sample Collection

**Figure 1:**
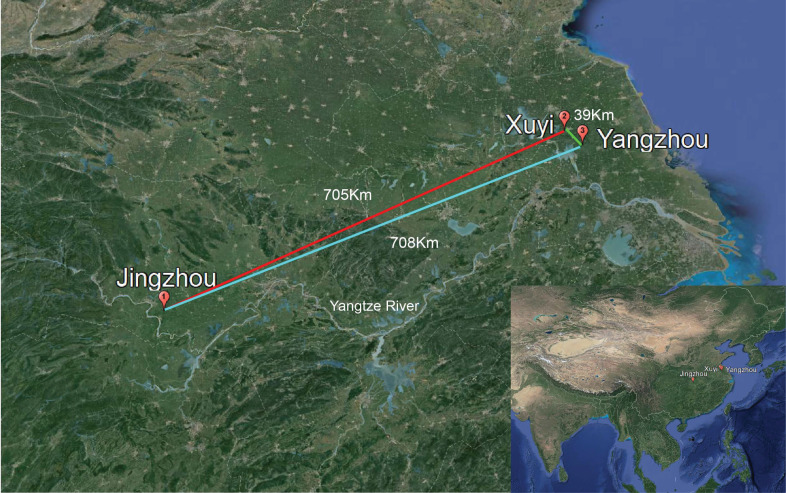
Sample locations: Jingzhou of Hubei province at the middle reaches of Yangtze river, and Yangzhou and Xuyi of Jiangsu province at lower reaches of Yangtze river. The distance between the sampling locations was indicated.

A total of 216 crayfishes and 180 eggs were collected in three regions of the two main breeding areas of China: Jingzhou (JZ), located in the middle reach of Yangtze River, Hubei province; and Yangzhou (YZ) and Xuyi (XY) located in the lower reaches of Yangtze River, Jiangsu province (Figure 1), in October, 2018. The crayfishes in Jingzhou and Xuyi were fed with fermented feed, while those in Yangzhou were fed with non-fermented feed. The larva, preadult, adult, berried crayfishes, and the eggs had an average (body) length of 4.53 ± 0.65 cm, 7.24 ± 0.38 cm, 9.56 ± 0.46 cm, 11.32 ± 0.33 cm and 0.21 ± 0.01 cm, and an average weight of 7.86 ± 0.23 g, 20.54 ± 0.36 g, 41.25 ± 0.29 g, 42.57 ± 0.51 g, and 0.03 ± 0.02 g, respectively. The crayfishes at above developmental stages were used for this study.

All crayfish dissections were performed under sterile conditions. Surgical tools were sterilized using 75% ethanol and flamed prior to use and between incisions. Gut dissections were carried out as follows: the gut samples were obtained by dissecting the crayfish, and were surface sterilized using 75% ethanol three times and rinsed three times in sterile phosphate-buffered saline (PBS). Then the gut samples were ground in a sterile grinder, and stored at −80 °C immediately until further processing. Every six crayfish intestines or 20 eggs from different locations or developmental stages were randomly mixed and extracted DNA as one sample. Every sample included three replicates.

### 2.2. DNA Extraction and 16S rRNA Gene Amplification

The V3-V4 region of the 16S rRNA genes was PCR amplified from a DNA aliquot of the extracted gut sample using the forward primer 341F (5′-CCTAYGGGRBGCASCAG-3′) and the reverse primer 806R (5′-GGACTACNNGGGTATCTAAT-3′) synthesized at Invitrogen (Invitrogen, Carlsbad, CA, USA). A unique 12 bp barcode to each primer was used to tag the samples. PCR reactions contained 25 μL 2 × Premix Taq (Takara, Dalian, China), 1 μL each primer (10 mM) and 3 μL DNA (20 ng/μL) template in a volume of 50 µL. They were amplified under the following conditions: 5 min at 94 °C for initialization; 30 s denaturation for 30 cycles at 94 °C, 30 s annealing at 52 °C, and 30 s extension at 72 °C; followed by 10 min final elongation at 72 °C in a Bio-Rad S1000 cycler. The length and concentration of the PCR product were detected using 1% agarose gel electrophoresis. The samples with bright main strip between 400–450 bp were used in further experiments. The PCR products were mixed in equidensity ratios according to GeneTools Analysis Software (Version4.03.05.0, SynGene). Thereafter, the mixed PCR products were purified with the EZNA Gel Extraction Kit (Omega, Norwalk, CT, USA).

### 2.3. High-Throughput Sequencing and Data Analysis

A total of 45 DNA samples were paired-end sequenced on Illumina HiSeq 2500 platform and 2 × 250 bp paired-end reads were generated. Firstly, clean reads were obtained through removing the adapter pollution and low-quality reads from raw data reads. The FastQC (http://www.bioinformatics.babraham.ac.uk/projects/fastqc/) and Trimmomatic (V0.33) tools were used to filter raw data reads to obtain high-quality clean reads [36]. The paired-end clean reads with overlaps were merged to clean tags using FLASH (V1.2.11) [37]. These tags were clustered into OTUs (operational taxonomic unit) using a 97% identity threshold and by Usearch software (V10) [38]. The representative sequences of each OUT were used to further annotation. The taxonomic information was annotated by GreenGene Database (http://greengenes.lbl.gov/). Data of OTUs abundance were normalized by the standard sample with the least sequences. Alpha diversity and beta diversity were analyzed by QIIME (V2.0, http://qiime.org/index.html).

The diversity of the gut bacterial community was compared to show the changes in crayfish developmental stages and geographic locations using Oneway Analysis of Variance (ANOVA) followed by Tukey post hoc test [39]. Non-Metric Multidimensional Scaling (NMDS) analyses and non-parametric multivariate analysis of variance (ADONIS) were performed to estimate the extent differences between (among) the groups, and the significance levels by calculating the weighted and unweighted UniFrac distance matrix using QIIME (V2.0). The alpha diversity was measured through four indices, including observed species, Chao1, Shannon, and Simpson with QIIME (V2.0) and visualized with R software (V3.0.3). To study the variance of the dominant species in different samples (groups), the OUT of representative sequence with the relative abundance in the first 50 and annotated to the level of the genus was selected. The multiple sequence alignment was conducted using the FastTree software [40]. The relative abundance of each OTU and the species annotation information of the representative sequence were combined using the ggtree software package for visualization. ANOSIM (Analysis of Similarity) on Bray–Curtis distance matrices was used to assess the relative contribution of three factors to the microbial community variation via the “anosim” function of the vegan package executed in R [41]. The factors included in the ANOSIM analysis comprised of development stage, diet, and geography. The percentage of variation described by each factor was estimated using R-value, and the significance (P-value) of each factor was obtained by 999 permutation tests.

## 3. Results

### 3.1. Dominant Bacteria at the Phyla Level

An Illumina Hiseq 2500 sequencing platform was utilized to analyze the structure of the crayfish gut microbiota. A total of 3,770,471 microbial 16S rRNA gene raw reads were assembled using FLASH at quality settings, obtaining 3,562,266 clean reads. The average sequencing reads of 45 samples was 57,163 reads per sample. For the further analyses, sequencing reads were normalized to minimum (35,792 reads) by randomly subsampling. After applying the Usearch clustering algorithm, all the 2493 unique OTUs were identified and allotted.

**Figure 2:**
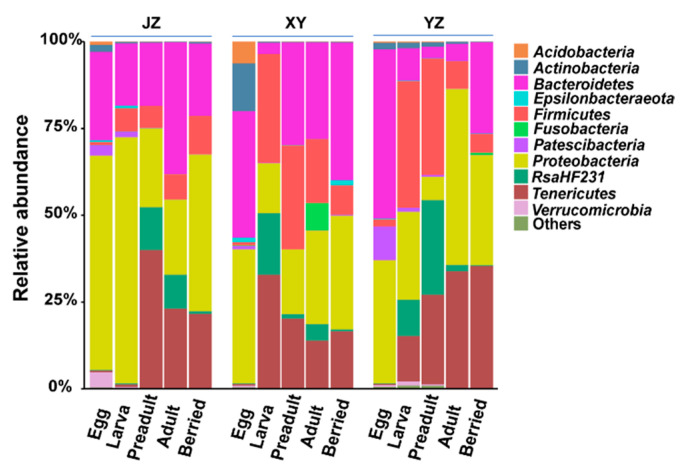
Relative abundances of the dominant bacteria at phyla level. *Proteobacteria*, *Bacteroidetes*, *Firmicutes*, *Tenericutes* and *RsaHF231* were most dominant across samples. JZ: Jingzhou, XY: Xuyi, and YZ: Yangzhou.

Across all the three different geographical regions, there were five predominant phyla, including *Proteobacteria*, *Bacteroidetes, Firmicutes*, *Tenericutes,* and *RsaHF231*. They had a higher than 5% abundances of the total sequences at the phylum level (Figure 2). To further identify the gut microbiota structure of the crayfish, the gut microbiota data was analyzed at the OTU level in each group. The relative abundances in the overall gut microbial community higher than 0.1% were selected for further analyses in each group. Although the 88 OTUs took up a minor part of all 2493 OTUs (3.53%) they contributed to more than 88.76% of the total sequences (Table S1). Interestingly, the 88 OTUs were allocated to the five dominant phyla and one unclassified bacteria OTUs (OTU4) were classified into *RsaHF231*.

### 3.2. Composition and Dynamics of Gut Microbiota during Developmental Stages

**Figure 3:**
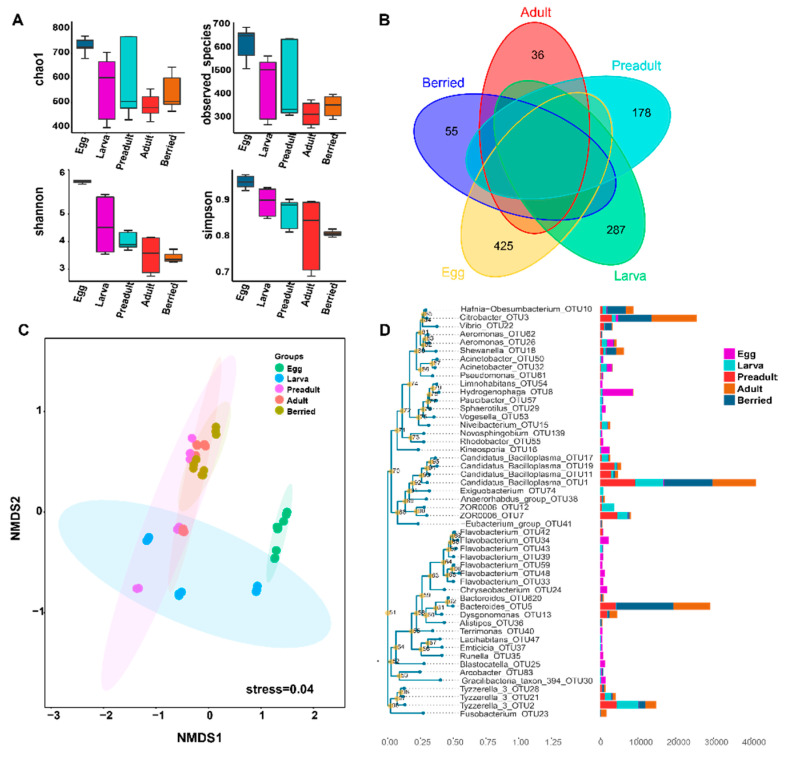
Trajectory of the gut microbiota in crayfishes across developmental stages. (**A**) The α-diverdity indexes (including observed species, Chao1, Shannon and Simpson) of crayfish during developmental stages. (**B**). Venn diagram showing OUT numbers change according to sample types. (**C**) Non-metric Multi-Dimensional Scaling ordination (NMDS) showing that bacterial communities cluster by sample types using the unweighted UniFrac distance. Adonis test, *R*^2^ = 0.382, *p* = 0.001. (**D**) The phylogenetic tree of top 50 OTUs relative to the relative abundance.

The dynamics of alpha diversity and gut microbial community structure were further studied. During the development, as exhibited by body length, weight, and shell color of the crayfishes, the Chao1 index and observed species reduced from 737 to 492, and 614 to 312, respectively (Figure 3A). Meanwhile, Shannon and Simpson indices reduced from 6.19 to 3.39, and 0.95 to 0.81, respectively (Figure 3A). Accordingly, the alpha diversity showed a declined trend during the crayfish development. Similarly, the Venn diagram showed a similar trend where the unique OUTs gradually reduced from 425 to 36 during the developmental stages (Figure 3B). The NMDS analysis based on weighted UniFrac distance revealed analogous gut microbiota clustering in a development-dependent manner (Figure 3C), indicating that development determines the gut microbiota in red swamp crayfish. These results were further corroborated by ANOSIM, revealing that the gut microbiota significantly (*p* < 0.05) differed between the two compared stages (Table 1). Particularly, the composition of the microbiota on the egg surface was different from any other stage (*p* < 0.05; Table 1). However, there was no significant difference between larva and preadult stages (*p =* 0.057; Table 1). The adult and berried stages had a similar microbial composition (*p =* 0.411; Table 1). According to Figure 3D, the genus and OTU level phylogenetic core microbial structure were shared in advanced crayfish developmental stages. The five genera including of *Candidatus_Bacilloplasma* (OTU1), *Tyzzerella_3* (OTU2), *Citrobacter* (OTU3), *Bacteroides* (OTU5), *Hydrogenophaga* (OTU8) were mainly dominant. *Bacteroides* (OTU5) were only distributed in the preadult and adult (berried) stages. *Hydrogenophaga* (OTU8) was unique in the early stages, and was mainly on egg samples. *Candidatus_Bacilloplasma* (OTU1) and *Tyzzerella_3* (OTU2) were found in the developmental stages except for the egg samples. *Citrobacter* (OTU3) was distributed in all stages.

### 3.3. Correlation between the Crayfish Gut Microbiota and Developmental Stages

**Figure 4:**
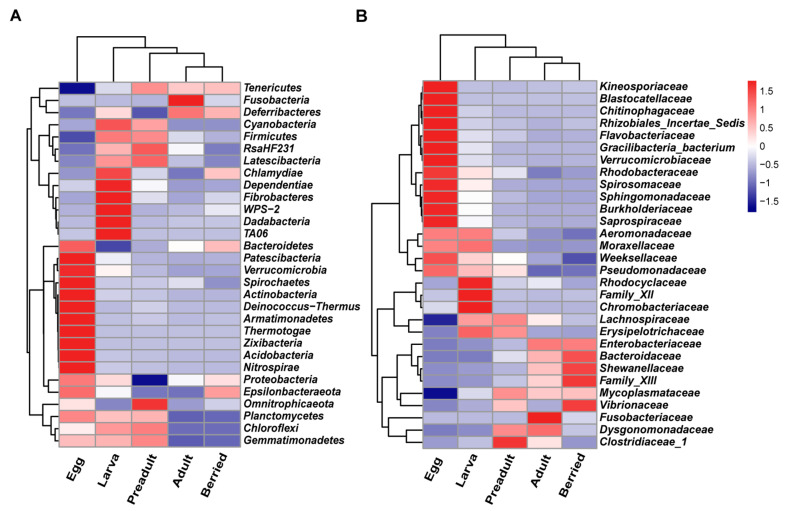
Heat map of the top 30 OTUs relative abundance at different developmental stages at the phyla level (**A**) or at the family level (**B**).

The composition of the first 30 families in the crayfish gut alternately changed and eventually stable during developmental stages (Figure 4A). The egg surface contained 15 unique phyla and shared 4 phyla with the larva stage, including *Proteobacteria, Planctomycetes, Chloroflexi,* and *Gemmatimonadetes*. The preadult stage shared seven phyla with the larval stage, and *Tenericutes* was only found in the adult stage (Figure 4A). Similarly, at the family level, the egg surface exclusively contained 16 unique families and shared four families with the larva stage, i.e., *Aeromonadaceae, Moraxellaceae, Weeksellaceae,* and *Pseudomonadaceae*. The high number of detected species, as well as unique phyla and families on the egg surface, are attributed to the crayfish laying their eggs to the abdomen where they are exposed to the environment (Figure 3B and Figure 4A,B). The preadult stage shared fewer bacteria with larval and adult stages (Figure 4B), suggesting susceptibility to the environment at this stage. However, the bacteria structures of the adult and berried stages were virtually constant (Figure 4).

**Figure 5:**
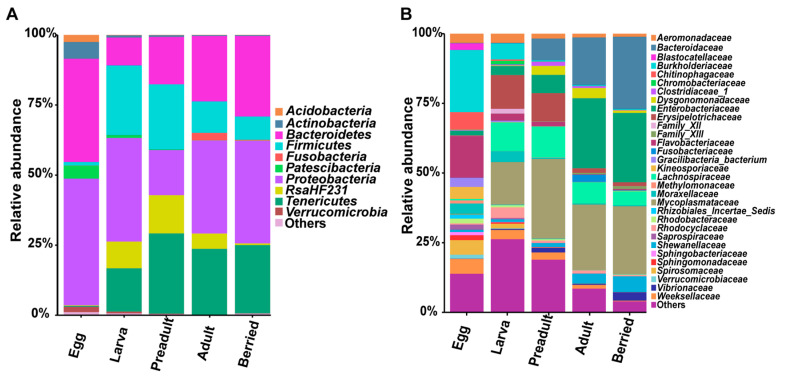
Dominant bacteria at different developmental stages. (**A**) Relative abundances of the dominant bacteria at the phyla level. (**B**) Relative abundances of the dominant bacteria at the family level.

Further, the core microbial communities in each group were analyzed at the phyla and family levels (Figure 5). Here, the relative abundance of *Proteobacteria* (45.20%) and *Bacteroidetes* (36.86%) accounted for 82.06% (Figure 5A). Specifically, *Firmicutes*, *Tenericutes,* and *RsaHF231* existed in each group except the egg group (Figure 5A). The *Bacteroidetes* significantly increased during the development (9.96% at the larva stage, 23.36% at the adult stage, and 28.75% at the berried stage). However, *Firmicutes* decreased from 24.84% at the larva to 11.20% at the adult stage and 8.30% at the berried stage. The *Proteobacteria* decreased and then increased during the developmental stages. The *Tenericutes* maintained a relatively stable situation. The bacteria with higher abundance (>2%) showed changes during developmental stages (Figure 5B). The families included *Burkholderiaceae* and *Flavobacteriaceae* dominated at the egg surface, and their relative abundances were 22.34% and 15.00%, respectively (Figure 5B). At the larval stage, the relative abundance of the bacterial communities higher than 5% were *Mycoplasmataceae* (15.33%), *Erysipelotrichaceae* (12.07%), *Lachnospiraceae* (10.31%), *RsaHF231* (9.53%), and *Burkholderiaceae* (5.79%). On the other side, the preadult stage mainly included *Mycoplasmataceae* (28.66%), *RsaHF231* (13.65%), *Lachnospiraceae* (11.12%), *Erysipelotrichaceae* (10.02%), *Bacteroidaceae* (7.75%), and *Enterobacteriaceae* (6.57%). The relative abundance of the *Bacteroidaceae* highly increased from 0.01% at the larval stage to 7.75% at the preadult stage. Besides, *Mycoplasmataceae* and *RsaHF231* increased from 15.33% to 28.66% and from 9.53% to 13.65%, respectively. At the adult or berried stage, the *Bacteroidaceae*, *Enterobacteriaceae*, and *Mycoplasmataceae* families were predominant in the gut region. Interestingly, *Bacteroidaceae* highly increased from 7.75% at the preadult stage to 17.03% (25.88%) at the adult (berried) stage, respectively (Figure 5B). Similarly, the *Enterobacteriaceae* highly increased from the preadult to the adult stage (Figure 5B). In summary, the relative abundance of the most dominant bacterial communities interchangeably varied during the developmental stages.

### 3.4. Effects of Diet and Geographical Location on Gut Microbiota

**Figure 6:**
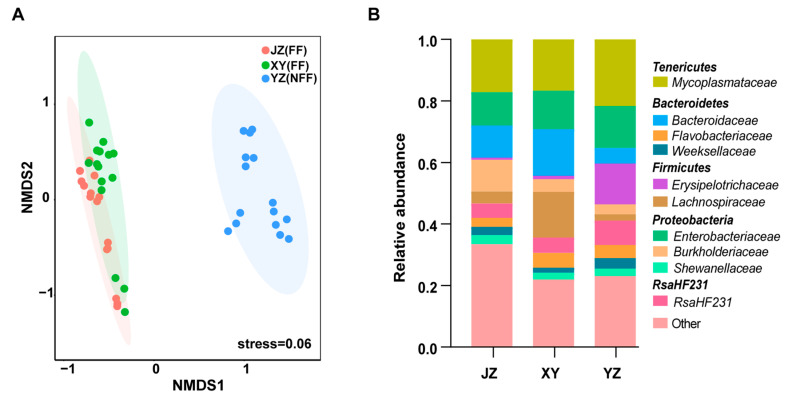
Impact of geography and diet on gut microbiota of crayfish. (**A**) Non-metric Multi-Dimensional Scaling ordination analysis of gut microbiota in crayfishes related to geographic locations. Adonis test, *R*^2^ = 0.122, *p* = 0.001. Red dots: gut samples of crayfishes from JZ fed with fermented feed; green dots: gut samples of crayfishes from XY fed with fermented feed; blue dots: gut samples of crayfish from YZ fed with non-fermented feed. (**B**) Relative abundances of the dominant bacteria in the guts of crayfish samples from different geographic locations (JZ: Jingzhou; XY: Xuyi; YZ: Yangzhou) at the family level.

The effects of diet and geography on the gut microbiota in red swamp crayfish were further studied. Crayfish in Jingzhou (JZ) and Xuyi (XY) were fed with the same fermented feed, while those in Yangzhou (YZ) were fed with non-fermented feed. Xuyi and Yangzhou are close, while Jingzhou is far away (Figure 1). Interestingly, the NMDS analysis based on unweighted UniFrac distance exhibited that the gut bacterial communities in the crayfish used the same diet from Jingzhou and Xuyi located far away from each other significantly gathered together (Adonis, *R*^2^ = 0.069, *p* = 0.08; Figure 6A). However, the gut bacterial communities in the red swamp crayfish from the closely located farms in Xuyi and Yangzhou expressively segregated into two groups (Adonis, *R*^2^ = 0.126, *p* = 0.024; Figure 6A). The ANOSIM analysis further corroborated that the gut microbiota significantly (*p* < 0.05) differed between the JZ and YZ, and the XY and YZ groups (Table 2). Furthermore, family classification demonstrated that JZ and XY groups had similar taxon (Figure 6B). Remarkably, the bacterial families with above 5% relative abundance in the three groups were similar (Figure 6B). The five most abundant families comprised of *Mycoplasmataceae*, *Enterobacteriaceae*, *Bacteroidaceae*, *RsaHF231*, and *Burkholderiaceae*. Their overall family-level phylogenetic core relative abundance was more than 50% (Figure 6B). However, *Erysipelotrichaceae* as a main family of *Firmicutes* phyla was widely spread in the YZ group (13.26%) and fewer in the JZ group (0.73%) and XY group (1.14%). ANOSIM based on the Bray–Curtis distance matrix was subsequently used in the assessment of the relative contribution of the environmental factors including, developmental stages, feeds, and geographic locations. The differences between the bacterial communities were mainly elucidated by the developmental stages (Table 3). According to these results, the developmental stage and then feeding are better determinants of gut bacterial composition than geography.

### 3.5. Fermented Feed Affects the Gut’s Microbial Composition

**Figure 7:**
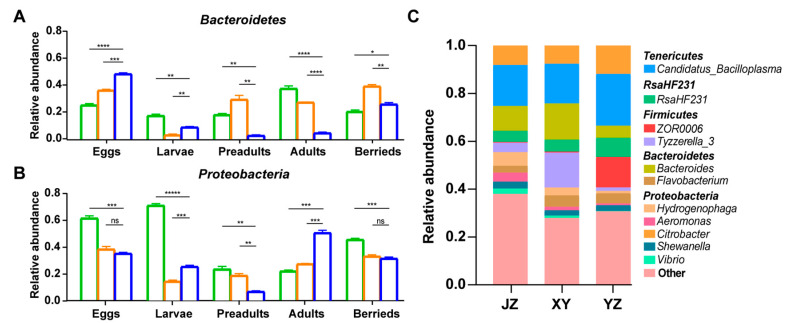
Changes of *Bacteroidetes* and *Proteobacteria* during developments. The relative abundance of *Bacteroidetes* (**A**) and *Proteobacteria* (**B**) phyla. Green bars: samples from Jingzhou; orange bars: samples from Xuyi; blue bars: samples from Yangzhou. (**C**) The composition of gut microbiota from three locations at the genus level. Significance: * *p* < 0.05, ** *p* < 0.01, *** *p* < 0.001, **** *p* < 0.0001, ***** *p* < 0.00001, one-way ANOVA.

Here, the effect of fermented and non-fermented feeds on the composition of gut microbiota in crayfish was compared at different developmental stages. It was revealed that *Bacteroidetes* were the dominant phyla at the surfaces of the eggs. Moreover, its abundance decreased at the larval stage in the guts of crayfishes at the three sampling locations (Figure 7A). The relative abundance of *Bacteroidetes* was significantly higher in the gut of crayfish fed with fermented feed than that fed with non-fermented feed in the preadult stage (JZ, XY, YZ: 18%, 28%, 3%; Figure 7A) and the adult stage (JZ, XY, YZ: 38%, 30%, 5%; Figure 7A). However, the relative abundance of *Bacteroidetes* increased in the berried stage of crayfishes fed with non-fermented feed (Figure 7A). The *Proteobacteria* also dominated in crayfish guts, but their relative abundance was significantly higher in the adult stage and significantly lower in the other stages of the crayfishes fed with non-fermented feed than fermented feed (Figure 7B). The analysis of the 11 most abundant genera revealed that *Bacteroides* was significantly higher (JZ, XY, YZ: 10.39%, 15.13%, 5.08%; Figure 7C) and *Citrobacter*, the possible pathogens, was lower (JZ, XY, YZ: 8.16%, 7.62%, 11.93%; Figure 7C) in the gut of crayfishes fed with fermented feed than in those fed with non-fermented feed during the development stage.

## 4. Discussion

Red swamp crayfish has become an economically important freshwater product in China. Regarded as the home of Chinese crayfish, Hubei and Jiangsu provinces are the main sources of crayfish in China. In 2018, more than 50% of the national yield was from this region especially Hubei province, constituting a major source of income for local farmers [28]. However, the incidence of disease outbreaks in the farmed species poses a great risk to the economic development during aquaculture environmental deterioration [19]. The use of probiotics and fermented food to promote host health by regulating the gut microbiota has been reported [42,43]. Therefore, studying the factors affecting the gut microbiota of red swamp crayfish is important in improving crayfish health and also the income to farmers.

In this study, *Proteobacteria*, *Bacteroidetes*, *Tenericutes*, *Firmicutes,* and *RsaHF231* constituted the predominant phyla. Specifically, *Proteobacteria* was the most dominant phyla in all the investigated samples. These results are consistent with the previous observation in the red crayfish (*P. clarkii*) [44] and other *Crustaceans*, such as the Pacific white shrimp (*L. vannamei*) [16,32], black tiger shrimp (*P. monodon*) [34], cherry shrimp (*N. denticulata*) [31], giant freshwater prawn (*M. rosenbergii*) [35], and Norway lobster (*N. norvegicus*) [30]. The *Proteobacteria* are dominant in aquatic invertebrate gut microbiotas of *Crustacea* and have high diversity in terms of physiology, morphology, and genetics [45]. However, the functions of *Proteobacteria* in *Crustacea* are not well understood. Besides, the *Bacteroidetes* and *Firmicutes* also constitutes the main members of a gut microbiota. Particularly, in healthy human adults, over 90% of microbiota are *Firmicutes* and *Bacteroidetes*, where they maintain a relatively stable condition and are involved in dietary plant polysaccharide metabolisms [46,47]. In this work, the *Firmicutes* genus in crayfish gut, *Tyzzerella_3* was detected in all samples. At the larval and preadult stages, *Tyzzerella_3* constituted 10% of the total gut microbiota. *Tyzzerella_3* may refer to involve growth and development factors so that the *Firmicutes* proportion is relatively high. However, *RsaHF231* was more likely to exist during the larval or preadult stages. As a *Candidatus*, *RsaHF231* has no cultured representatives and exist in some species, such as black soldier fly (*Hermetia illucens*), *Culex* mosquitoes, red swamp crayfish (*P. clarkii*) and Atlantic salmon (*Salmo salar*) [44,48,49,50,51]. There no relevant report performed *RsaHF231* phylum distributed in other species. Interestingly, *RsaHF231* has been found in Atlantic salmon that was feed black soldier fly larvae meal. The reason may have partially originated from black soldier fly larvae meal [51]. For crayfish, *RsaHF231* also has been found in gut microbial. It seems to be a transient species from the environment and its relative abundance decreased with development. In terms of the biological significance of phylum *RsaHF231*, there is still no reasonable and significant explanation and further research is needed. Notably, *Tenericutes* has been detected at later developmental stages. At the genus level, *Candidatus Bacilloplasma* was dominant with *Tenericutes* being significantly high abundant. Throughout its lifetime, *Candidatus Bacilloplasma* maintained a high relative abundance. “*Candidatus Bacilloplasma*” has been associated with the gut surface of *Porcellio scaber* and it plays a role in the digestion process [52]. This indicates that these bacteria are commensals and well adapted to the crayfish gut environment.

Furthermore, a remarkable change in red crayfish gut microbial composition was found during the developmental stage. At the early development stages (larva and preadult) the gut microbial composition diversity was the highest. This may be attributed to the weak selection exerted by the larvae on exogenous colonizers due to the immature gut (e.g., unoccupied niche), resulting in relatively stochastic assembly [53]. Thus, it is likely that the age of the host generally affects the gut microbiota. Since crayfish is omnivorous, the age discrepancy in the gut microbial structure is mainly associated with individual developmental stage and dietary changes. In early stages (during larva and preadult), the larva has not yet developed into a mature claw so they can only prey on small-grain feed and algae. However, an adult crayfish can use strong claw to capture food including plant-derived and animal feed as well as the larva crayfish and dead aquatic organisms. Besides, a possible cause of gut microbial variation is that *Crustacea* guts are unstable habitats due to the occurrence of several molts during development [54]. It is common for terrestrial isopods to ingest the shed cuticle, including the hindgut cuticle after molting to restore lost minerals. This is a possible way of gut recolonization after molting [52]. Crayfish can regain these microbes, thus there is microbial alternating during development and some bacterial communities are passed from the larval stages to the adult stage. Nevertheless, the relationship between the molting process and gut microbial community still needs to be further studied. Moreover, variations of the gut bacterial community from larvae to adults can lead to drastic dietary changes. For *Actinobacteria*, they were very few in crayfish gut in this study. These results differ from a recent study, where crayfishes sampled in rice fields were rich in plant biomass but not feed [44]. *Actinobacteria* are mainly regarded as free-living microorganisms that play a critical role in the breakdown of plant biomass [55]. Accordingly, diet strongly affects the gut microbiota of red swamp crayfish. The egg surface had a significant discrepancy from the other stages and higher diversity of microbes. This is probably because the eggs were exposed in water and the microbial communities from the environment.

Here, the fermented feed significantly increased *Bacteroidetes* and decreased *Proteobacteria* in adults (Figure 7A,B). The *Bacteroides* group is one of the most important groups in the intestinal microbiota of the *Crustacea* [23]. They have contained many genes involved in polysaccharide and monosaccharide metabolism, thus important for nutrient absorption in crayfish [56,57]. In addition, *Bacteroides* produce propionate, which can ameliorate colitis thus enhancing the intestinal barrier function and reducing inflammation [58]. The fermented feed contained soybean, corn, and wheat hence supplying more polysaccharides and probiotics (yeast and lactic acid bacteria) than the basal diet. Therefore, this can be attributed to an increase in *Bacteroidetes* in the gut microbiota of crayfish fed on fermented feed supplemented diets. In other aquatic animals, studies found that fermented feed can promote host health. Study found fermented soybean meal through *Bacillus pumillus* SE5 and *Pseudozyma aphidis* ZR1 fermentation beneficially influences feed utilization, antioxidant capacity, innate immunity and gut health in juvenile Japanese seabass [59]. This study suggests that fermented feed (autolyzed yeast replace fish meal) can regulate gilthead sea bream gut microbial composition. The abundance of some beneficial bacteria, i.e., indigestible carbohydrate degrading and SCFA producing microbial, was positively affected [60]. Therefore, fermented feed has a great positive effect on fish gut microbiota. Besides, among the two diets, there was a lower relative abundance of *Proteobacteria* and the genus *Citrobacter* in the fermented feed group, and a higher relative abundance in the non-fermented feed group in adults. Importantly, most of *Proteobacteria* are considered as opportunistic pathogens and exist harmoniously with host, such as *Vibrio* spp. are often described as the dominant genus in gut microbiota of shrimp and live in association with shrimp [45]. Certainly, when the relative abundance of these disease-related *Proteobacteria* widely changed and increased, it may indicate the change may pose a threaten to host health. In this study, *Citrobacter* genus belong to *Proteobacteria* phylum has a high relative abundance in non-fermented feed group in adults compared with other groups. It suggested the crayfish was in an unhealthy state though health situation cannot be judged by physical appearances. As an opportunistic pathogen, *Citrobacter* mainly causes significant opportunistic infections in humans [61]. The *Citrobacter* pathogenic strains can also cause septicemia and death in aquatic organisms such as *Rhamdia quelen* [62]. It is speculated that *Citrobacter* can cause disease in the crayfish. Accordingly, this result suggests that fermented feed is also important for resisting pathogenic bacteria. Moreover, other genera from *Proteobacteria*, *Aeromonas*, *Shewanella,* and *Vibrio* have been considered as opportunistic pathogens in aquatic environments [63]. Whether fermented feed or probiotics can help alter the gut microbiota of red swamp crayfish need to be further investigated.

## 5. Conclusions

Overall, the results revealed that development, followed by diet, are the most prominent factors that determine gut bacterial composition compared to geography. In addition, it was evident that fermented feed is important in nutrient absorption where it increases the relative abundance of *Bacteroides* in the gut of red swamp crayfish.

## Figures and Tables

**Table 1:** Community dissimilarity test of gut microbiota over crayfish developmental stages based on analysis of similarity (ANOSIM) using Bray–Curtis distance.

Stage	Egg		Larvae		Preadult		Adult		Berried	
Egg	-	-								
Larvae	0.725	**0.002**	-	-						
Preadult	1.000	**0.001**	0.147	0.057	-	-				
Adult	1.000	**0.001**	0.420	**0.001**	0.189	**0.034**	-	-		
Berried	1.000	**0.001**	0.632	**0.001**	0.439	**0.001**	0.004	0.411	-	-

On the left is *R* value indicating the correlation. On the right is *p* value indicating significant differences. Bold values represent significant differences (*p* < 0.05) between pairwise stages.

**Table 2:** Community dissimilarity test of gut microbiota over crayfish diet and geography based on analysis of similarity (ANOSIM) using Bray–Curtis distance.

Group	Jingzhou		Yangzhou		Xuyi	
Jingzhou	-	-				
Yangzhou	0.126	**0.028**	-	-		
Xuyi	0.069	0.101	0.204	**0.002**	-	-

On the left is *R* value indicating the correlation. On the right is *p* value indicating significant differences. Bold values represent significant differences (*p* < 0.05) between pairwise stages.

**Table 3:** ANOSIM of crayfish gut microbiota based on Bray–Curtis distance calculated with partial 16S rRNA sequences.

Factor	*N*	*R*	*p*
Age	5	0.561	0.001
Diet	2	0.204	0.002
Geography	3	0.144	0.001

*N* value indicated the number of groups; *R* value indicated the correlation; *p* value indicated significant differences (*p* < 0.05).

## Supplementary Materials

The following are available online at https://www.mdpi.com/2076-2607/8/9/1376/s1. Table S1: Summary of OUT level relative abundance more than 1%.

## Data Accessibility

Molecular sequence data reported in this paper have been deposited in the National Center for Biotechnology Information (NCBI) Sequence Read Archive (SRA accession: PRJNA609648).
